# Kennwerte und teststatistische Güte des *Veterans RAND 12-Item Health Survey *(VR-12) bei Patienten mit chronischem Schmerz: Eine Auswertung auf Basis des KEDOQ-Schmerz-Datensatzes

**DOI:** 10.1007/s00482-021-00570-5

**Published:** 2021-07-19

**Authors:** M. Hüppe, K. Schneider, H.-R. Casser, A. Knille, T. Kohlmann, G. Lindena, B. Nagel, J. Nelles, M. Pfingsten, F. Petzke

**Affiliations:** 1 Klinik für Anästhesiologie und Intensivmedizin, Universität zu Lübeck, Ratzeburger Allee 160, 23538 Lübeck, Deutschland; 2 DRK Schmerz-Zentrum Mainz, Mainz, Deutschland; 3 Schmerzmedizin, Rheinland Klinikum, Standort Grevenbroich, Grevenbroich, Deutschland; 4 Institut für Community Medicine, Universität Greifswald, Greifswald, Deutschland; 5 CLARA Klinische und Versorgungsforschung Kleinmachnow, Kleinmachnow, Deutschland; 6 Klinik für Schmerzmedizin, St. Vincenz Hospital Brakel, Brakel, Deutschland; 7 Schmerzklinik, Universitätsmedizin Göttingen, Göttingen, Deutschland

**Keywords:** Deutscher Schmerzfragebogen, PROMs, Gesundheitsbezogene Lebensqualität, Qualitätssicherung, KEDOQ-Schmerz, German Pain Questionnaire, Patient Reported Outcome Measures, Health-related quality of life, Quality assurance, KEDOQ pain

## Abstract

Der *Veterans RAND 12-Item Health Survey* (VR-12) wurde als Selbstbeurteilungsverfahren zur Erfassung gesundheitsbezogener Lebensqualität 2016 in den Deutschen Schmerzfragebogen (DSF) aufgenommen und ersetzt den bis dahin verwendeten lizenzpflichtigen SF-12. Beide Verfahren haben 12 Items und führen zu einer körperlichen und einer psychischen Summenskala. Auswertungen der deutschen Version mit größerer Stichprobe zu Kennwerten und zur teststatistischen Güte des VR-12 bei Patienten mit chronischem Schmerz fehlen bislang.

Daten zum VR-12 und weiterer Verfahren des DSF wurden von 11.644 Patienten aus 31 an KEDOQ-Schmerz beteiligten Zentren ausgewertet. Die Patienten hatten den DSF vor Beginn einer angestrebten schmerztherapeutischen Behandlung ausgefüllt. Die Bestimmung der Änderungssensitivität erfolgte für 565 Patienten, für die der VR-12 aus einem Verlaufsfragebogen des DSF mehrere Monate nach der Ersterhebung vorlag.

Die Reliabilität (Cronbachs Alpha) der körperlichen Summenskala war *r*_*tt*_ = 0,78 und für die psychische Summenskala *r*_*tt*_ = 0,84. Die psychische Summenskala wies deutliche Beziehungen zu den Depression-Angst-Stress-Skalen (DASS) auf (*r* = −0,51 bis r = −0,72), die körperliche Summenskala korrelierte höher mit Bereichen schmerzbedingter Beeinträchtigungen (*r* = −0,48 bis *r* = −0,52). Patienten mit höherer Schmerzchronifizierung, solche mit höherem Schmerz-Schweregrad und solche mit Hinweis auf hohe psychische Belastung beschrieben in beiden Summenskalen signifikant geringere gesundheitsbezogene Lebensqualität. Die Effektstärke (ES) der Veränderung im Sinne einer Verbesserung der gesundheitsbezogenen Lebensqualität war in der psychischen Summenskala ES = 0,33 und in der körperlichen Summenskala ES = 0,51.

Die Ergebnisse sind in Übereinstimmung zu Befunden zum SF-36 bzw. SF-12 bei anderen Patientenkollektiven mit chronischem Schmerz. Sie zeigen zusammengefasst, dass mit dem VR-12 ein angemessener Ersatz zum SF-12 im Deutschen Schmerzfragebogen integriert wurde.

**Zusatzmaterial online:**

Die Online-Version dieses Beitrags (10.1007/s00482-021-00570-5) enthält den VR-12-Fragebogen und eine Auswertungsanleitung.

## Abstract (en)

The Veterans RAND 12-Item Health Survey (VR-12) was added to the German Pain Questionnaire (DSF) as a self-report measure of health-related quality of life in 2016, replacing the previously used SF-12, which required a license. Both measures have 12 items and include a physical component summary (PCS) and a mental component summary (MCS). Evaluations with a larger sample on characteristic values and on the test-statistical goodness of the VR-12 in patients with chronic pain are so far missing.

Data on the VR-12 and other procedures of the DSF were evaluated from 11,644 patients from 31 centers participating in KEDOQ pain. The patients filled out the DSF before starting a pain therapy treatment. Change sensitivity was determined for 565 patients for whom the VR-12 was available from a follow-up questionnaire of the DSF several months after the initial survey.

The reliability (Cronbach’s alpha) of the PCS was *r*_*tt*_ = 0.78 and for the MCS *r*_*tt*_ = 0.84. The MCS had significant relationships with the depression, anxiety and stress scales (*r* = −0.51 to *r* = −0.72), and the PCS correlated more highly with areas of pain-related impairment (*r* = −0.48 to *r* = −0.52). Patients with higher pain chronicity, those with higher pain severity, and those with evidence of high psychological distress described significantly lower health-related quality of life in PCS and MCS. The effect size (ES) of change in terms of improvement in health-related quality of life was ES = 0.33 in the MCS and ES = 0.51 in the PCS.

The results are in agreement with the findings of the SF-36 and SF-12 in patient collectives with chronic pain. In summary, they show that the VR-12 is an adequate substitute for the SF-12 in the German pain questionnaire.

## Einleitung und Fragestellung

Chronische Schmerzen, für die Patienten Behandlungen ersuchen, sind mit reduzierter gesundheitsbezogener Lebensqualität assoziiert [7, 9, 12, 13, 22], und für die Erfassung des Erfolgs einer schmerztherapeutischen Behandlung wird eine Verbesserung der gesundheitsbezogenen Lebensqualität als *ein* zentraler Outcome-Parameter empfohlen [15].

Der *Veterans RAND 12-Item Health Survey* (VR-12) ist ein Messinstrument zur Bestimmung von gesundheitsbezogener Lebensqualität [1, 2, 16, 18]. Die Deutsche Schmerzgesellschaft hat den VR-12 im Rahmen des Deutschen Schmerzfragebogens (DSF) 2016 als Instrument eingeführt und damit den lizenzgebundenen Short-Form 12 Item Health Survey (SF-12) ersetzt. Damit gehört der VR-12 zu den „jüngeren“ psychometrischen Verfahren des DSF, dessen Handanweisung bislang keine Angaben zu teststatistischen Kennwerten des Verfahrens beinhaltet [24].

Hinsichtlich Umfang und Merkmalsbereichen sind VR-12 und SF-12 vergleichbar. Die 12 Items des VR-12 werden wie beim SF-12 in zwei Summenskalen für die körperliche und die psychische Gesundheit verrechnet. Hohe Werte in den Summenskalen entsprechen einer hohen Ausprägung der körperlichen und psychischen Gesundheit. In jede Summenskala geht die Berechnung aller 12 Items ein. Dabei werden 6 Items für die *körperliche Summenskala* und 6 Items für die *psychische Summenskala* entsprechend ihrer inhaltlichen Beziehung zu den Summenskalen höher gewichtet. Vergleichbar dem SF-12 sind die Summenskalen des VR-12 durch die Wahl der Gewichte und einer skalenspezifischen Konstante mit einem Mittelwert von 50 und einer Standardabweichung von 10 normiert. Die entsprechenden Gewichte und Konstanten liegen bislang aus einer repräsentativen (US-)Population vor [26]. Werte von 40 oder weniger kennzeichnen damit Personen mit einer geringen gesundheitsbezogenen Lebensqualität, die mindestens eine Standardabweichung unter der Bevölkerungsnorm liegt.

Seit 2015 existiert der VR-12 in deutscher Sprache (siehe elektronisches Zusatzmaterial). Die deutschsprachige Übersetzung, Adaptation und Validierung wurde von der Abteilung Methoden der Community Medicine der Universität Greifswald in Zusammenarbeit mit der Boston University vorgenommen [2, 14, 16, 17]. Die Validitätsüberprüfung des VR-12 erfolgte anhand unterschiedlicher Stichproben hauptsächlich im angloamerikanischen Raum.

In Deutschland verglichen Buchholz, Kohlmann und Buchholz [1] die Verteilungseigenschaften, Testgüte und Änderungssensitivität des SF-36/SF-12 mit denen des VR-36/VR-12 an Patienten, die eine stationäre orthopädische oder psychosomatische Rehabilitationsmaßnahme erhielten. Die Autoren fassen als Ergebnis ihrer Analysen zusammen, „dass sich die deutschsprachige Version des VR als ein dem SF-Fragebogen gleichwertiges Instrument in der hier untersuchten Population erwies“ (S. 46).

Auswertungen zu Kennwerten und zur teststatistischen Güte der deutschen Version des VR-12 bei Patienten mit chronischem Schmerz fehlen bislang aus größeren Stichproben. Unsere Analyse soll diese Lücke schließen. Dabei werden Daten zum VR-12 und weiterer Verfahren des DSF (Handbuch Petzke et al. [24]) ausgewertet, die im KEDOQ-Schmerz-Kerndatensatz hinterlegt sind [4].

KEDOQ-Schmerz ist ein Projekt der Deutschen Schmerzgesellschaft und hat den Anspruch einer umfassenden Dokumentation schmerztherapeutisch versorgter Patienten im ambulanten, teilstationären und stationären Behandlungssektor [4, 12, 13]. Ausgewählte Daten aus diesen Einrichtungen werden zu einem Kerndatensatz zusammengeführt. Ziel ist ein Register aus patientenbezogenen Merkmalen sowie diagnose- und therapierelevanten Daten, um damit längerfristig eine valide und repräsentative Charakterisierung schmerztherapeutisch versorgter Patienten zu Beginn der Behandlung, im Behandlungsverlauf und katamnestisch für Aussagen zur Nachhaltigkeit der schmerztherapeutischen Versorgung zu ermöglichen.

Der KEDOQ-Schmerz-Datensatz besteht aus Angaben aus dem *Deutschen Schmerzfragebogen,* aus Angaben zum Behandlungssetting sowie aus weiteren Informationen zum Schmerz und zur Vorbehandlung. Der Behandlungsverlauf wird mittels Verlaufsfragebögen des DSF und Informationen zur Behandlungsintensität und -qualität begleitet, auch der Status bei (teil-)stationärem Behandlungsabschluss wird damit dokumentiert. Die Katamneseerhebungen erfolgen drei bis sechs Monate nach Behandlungsbeginn, ebenfalls dokumentiert mit dem Verlaufsfragebogen des DSF.

## Methodik

Nach positivem Votum des Ethikrats der Deutschen Schmerzgesellschaft (05.11.2020) und Zustimmung zu einer Anzeige bei der Ethikkommission der Universität zu Lübeck (AZ 20-422) wurden für die Analyse Daten aus dem KEDOQ-Schmerz-Register von Patienten ausgewertet, die den Deutschen Schmerzfragebogen zwischen Januar 2014^1^ und November 2020 ausgefüllt hatten und für die Angaben zum VR-12 vorlagen. Die Patienten hatten den DSF vor Beginn einer angestrebten schmerztherapeutischen Behandlung ausgefüllt.

Aus dem KEDOQ-Schmerz-Datensatz wurden folgende Variablen bzw. Merkmale zur Datenanalyse herangezogen:

- Soziodemografische Angaben: Alter, Geschlecht, Schulabschluss, Berufstätigkeit
- Schmerzbezogene Parameter: Schmerzdauer, Schmerzintensität (numerische Rating Skala [NRS: 0 = kein Schmerz, 10 = stärkster vorstellbarer Schmerz] zur Erfassung der durchschnittlichen, maximalen und aktuellen Schmerzintensität), schmerzbedingte Beeinträchtigung in drei Lebensbereichen (Alltag, Freizeit, Arbeit), Anzahl der Tage schmerzbedingter Beeinträchtigungen sowie der aus diesen Angaben gebildete Schmerz-Schweregrad nach von Korff [27].
- Psychometrische Daten: Fragebogen zur Erfassung gesundheitsbezogener Lebensqualität (VR-12 [1, 18]); Depression-Angst-Stress-Skalen (DASS [21, 23]); Marburger Fragebogen zum habituellen Wohlbefinden (MFHW [10]).
- Zusatzinformationen (Arztangaben) lagen zum Schmerzchronifizierungsstadium (Mainz Pain Staging System, MPSS [8]), zum Hauptschmerz sowie zum Behandlungssetting (ambulante, teilstationäre oder stationäre Behandlung) vor. Der Hauptschmerz wurde klassifiziert nach der Schmerzlokalisation sowie der medizinischen Hauptschmerzdiagnose.

Die Daten wurden in anonymisierter Form von dem für KEDOQ-Schmerz beauftragten Institut CLARA zur Verfügung gestellt.

Zur Bestimmung der Kennwerte des VR-12 wurden Lage und Streuparameter berechnet und zur Veranschaulichung der Verteilung Histogramme erstellt. Zur Prüfung der Normalverteilung der Summenskalen wurde der Kolmogorov-Smirnov-Test gerechnet.

Die Bestimmung der Reliabilität der zwei Summenskalen des VR-12 erfolgte über Berechnungen zur internen Konsistenz (Cronbachs Alpha). Die unterschiedlichen Antwortkategorien der einzelnen Items (3–6 Antwortkategorien) wurden für diese Berechnung entsprechend den Auswertungsvorgaben zum VR-12 in Werte zwischen 0 und 100 transformiert. Hohe Werte entsprechen hoher gesundheitsbezogener Lebensqualität. Für die interne Konsistenzbestimmung der zwei Summenskalen wurden jeweils die Items verwendet, die in den Skalen des VR-36 der körperlichen oder psychischen Summenskala zugeordnet sind.

Zur Validitätsbestimmung wurden Produkt-Moment-Korrelationen zwischen den zwei Summenskalen des VR-12 und den Skalen der DASS (Depression-Angst-Stress-Skalen), dem MFHW (Marburger Fragebogen zum Habituellen Wohlbefinden) sowie den drei Skalen zu schmerzbedingten Beeinträchtigungen berechnet. Die Interpretation der Höhe der Korrelationen erfolgte nach Cohen [5] (*r* ≥ 0,10: geringer Zusammenhang, *r* ≥ 0,30: mittelstarker Zusammenhang; *r* ≥ 0,50: starker Zusammenhang).

Für klassifizierbare Merkmale (Schmerz-Schweregrad, Chronifizierungsstadium, DASS-Skalen mit Cut-off-Werten für psychische Belastung) wurden Gruppenvergleiche (*t*-Tests oder Varianzanalysen mit Tukey-Folgetest bei signifikantem Globaleffekt) berechnet.

Für die Bestimmung der Änderungssensitivität wurden die bei Behandlungsbeginn erhobenen Werte mit denen verglichen, die am nächsten an einer sechsmonatigen Follow-up-Messung lagen. Veränderungen wurden als Effektstärken formuliert. Die Berechnung der Effektstärke zur Kennzeichnung der Änderungssensitivität erfolgte für Prä-Post-Design mit einer Gruppe unter Berücksichtigung der Korrelation der zwei Messungen bei Bildung der verwendeten Standardabweichung. Dies hat zur Folge, dass die Abhängigkeit der Messungen und damit das mathematisch korrekte Verfahren zur Berechnung der Varianz von Erst- und Verlaufsmessung Berücksichtigung findet [5].

Effektstärken ab *d* ≥ 0,20 wurden als „kleiner Effekt“, solche ab *d* ≥ 0,50 als „mittlerer Effekt“ und die ab *d* ≥ 0,80 als „großer Effekt“ interpretiert [5].

Die Berechnungen zur Effektstärke der Änderungssensitivität erfolgten mit dem Programm von Lenhard und Lenhard [20]. Die weiteren Analysen wurden mit SPSS (Version 22) durchgeführt.

## Ergebnisse

Von 11.644 Patienten aus 31 an KEDOQ-Schmerz beteiligten Zentren lagen aus dem DSF Angaben zum VR-12 vor. Diese Gruppe bildete die Analysestichprobe. Die Patienten hatten ein Durchschnittsalter von 55,5 (*SD* = 14,9) Jahren und waren mehrheitlich weiblich (66,7 %). Die meisten Patienten hatten einen Haupt- oder Realschulabschluss (70,6 %) und nahezu die Hälfte hatte den DSF vor einer stationären Behandlung ausgefüllt (49,5 %). Etwa die Hälfte der Patienten war berufstätig (48,7 %).

Die Hauptschmerzlokalisation betraf überwiegend die Lendenwirbelsäule (Kreuz/Bein, 30,9 %) und existierte seit mindestens zwei Jahren, (63,9 %). Die meisten Patienten hatten den höchsten Schmerz-Schweregrad nach v. Korff (Stufe 4, 63,7 %) und das höchste Schmerzchronifizierungsstadium nach dem Mainzer Stadienmodell (Stadium III: 57,0 %).

Tab. 1 fasst einige Patientenmerkmale zusammen.

**Table 1:** 

Merkmal *N* = 11.644	Ausprägung
*Geschlecht [n (%)]*	
Männer	3872 (33,3)
Frauen	7772 (66,7)
*Alter in Jahren [M (SD)] N* = 11.635	55,5 (14,9)
*Schulabschluss [n (%)]*	
Kein Abschluss	443 (3,8)
Hauptschule	4400 (37,8)
Realschule	3822 (32,8)
Fachabitur	1057 (9,1)
Abitur	1922 (16,5)
*Berufstätigkeit [n (%)]*	
Ja	5667 (48,7)
Nein	5977 (51,3)
Behandlungsanlass [*n* (%)]	
Ambulant	4159 (35,7)
Teilstationär	1716 (14,7)
Stationär	5769 (49,5)
*Hauptschmerz [n (%)]*	
1 Kopf/Gesicht	545 (4,7)
2 Nacken/Schultergürtel/Arm	1093 (9,4)
3 Rücken/BWS	270 (2,3)
4 Kreuz/Bein	3599 (30,9)
5 Extremitäten	1536 (13,2)
6 Abdomen/Genitalregion	153 (1,3)
7 ausgedehnte Schmerzbilder	1161 (10,0)
8 Sonstige Schmerzverteilung	995 (8,5)
9 Becken/Bein	237 (2,0)
10 WS mehrere Lokalisationen	295 (2,5)
11 mehr als 3 Lokalisationen	696 (6,0)
12 keine Zuordnung	1064 (9,1)
*Schmerzdauer [n (%)]*	
< 1 Jahr	2608 (22,4)
1–5 Jahre	4097 (35,2)
> 5 Jahre	4939 (42,5)
*Schmerzchronifizierung (MPSS) [n (%)]*	
Stadium I	835 (7,2)
Stadium II	4168 (35,8)
Stadium III	6641 (57,0)
*Schmerz-Schweregrad [n (%)]*	
Grad 1	481 (4,1)
Grad 2	1299 (11,2)
Grad 3	2437 (20,9)
Grad 4	7420 (63,7)

### Statistische Kennwerte, Verteilungseigenschaften und Reliabilität des VR-12

**Figure 1:**
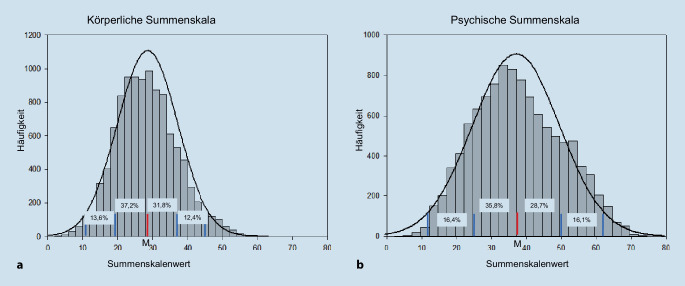

Für die Gesamtgruppe wies die körperliche Summenskala einen Mittelwert von 28,2 (*SD* = 8,7) und einen Median von 27,7 auf. Der Mittelwert der psychischen Summenskala war 37,6 (*SD* = 12,6) und der Median 37,6. Beide Verteilungen wichen signifikant von einer Normalverteilung ab (*p* < 0,001 für beide Summenskalen). Abb. 1 zeigt die Histogramme für beide Summenskalen, deren Schiefe eine linkssteile Verteilung anzeigt (körperliche Summenskala: Schiefe = 0,326; psychische Summenskala: Schiefe = 0,197). Die Kurtosis war für die körperliche Summenskala 0,082, was einer Normalverteilung gut entspricht, und die Kurtosis der psychischen Summenskala war −0,588, was schwächer ausgeprägte Randbereiche im Vergleich zur Normalverteilung nahelegt. Für die körperliche Summenskala lagen 69,0 % der beobachteten Werte im Bereich M ± 1 SD, 95,0 % im Bereich M ± 2 SD und 99,7 % im Bereich M ± 3 SD. Für die psychische Summenskala waren die entsprechenden Werte 64,5 %, 97,0 % und 100 %, bei einer „idealen“ Normalverteilung lägen sie bei 68 %, 95 % und 99 %. Für beide Summenskalen ergaben sich etwas mehr Beobachtungen links vom arithmetischen Mittel (körperliche Summenskala: 52,4 %, psychische Summenskala: 52,8 %).

Die zwei Summenskalen waren statistisch nahezu unabhängig (*r* = −0,024).

Tab. 2 fasst für die Gesamtgruppe und die Behandlungssektoren statistische Kennwerte für die Summenskalen des VR-12 zusammen.

**Table 2:** 

Behandlungssektor	Ambulant (*n* = 4159)	Teilstationär (*n* = 1716)	Stationär (*n* = 5769)	Gesamt (*n* = 11.644)
*Körperliche Summenskala*				
Mittelwert	28,32	30,73	27,38	28,21
Standardabweichung	8,90	8,52	8,37	8,66
Minimum-Maximum	3,3–59,5	3,9–60,2	3,3–59,9	3,3–60,2
Perzentil 25	22,06	24,87	21,40	22,10
Median	27,88	30,60	26,80	27,70
Perzentil 75	33,90	36,62	32,70	33,65
*Psychische Summenskala*				
Mittelwert	37,97	38,91	36,85	37,55
Standardabweichung	12,77	11,82	12,61	12,58
Minimum-Maximum	6,0–72,0	8,3–68,8	2,2–77,0	2,2–77,0
Perzentil 25	28,30	30,46	27,40	28,20
Median	37,29	38,41	35,80	36,63
Perzentil 75	47,44	47,60	45,85	46,70

Anmerkung: hohe Werte entsprechen hoher gesundheitsbezogener Lebensqualität

Für die Reliabilitätsberechnung konnten nur die Patienten berücksichtigt werden, für die die Einzelitems des VR-12 verfügbar waren (*n* = 10.722). Für die übrigen 922 Patienten lagen ausschließlich die Summenwerte vor. Cronbachs Alpha war für die körperliche Summenskala *r*_*tt*_ = 0,782 und für die psychische Summenskala *r*_*tt*_ = 0,842.

### Beziehungen des VR-12 mit konstruktverwandten Tests und Skalen schmerzbedingter Beeinträchtigung

Zur Prüfung der Validität wurden die zwei Summenskalen des VR-12 mit konstruktnahen Skalen der DASS (Angst, Depression, Stress), dem MFHW (Habituelles Wohlbefinden) und den drei Skalen zur schmerzbedingten Beeinträchtigung in Beziehung gesetzt. Tab. 3 zeigt die Korrelationen.

**Table 3:** 

Verfahren (*n*)	Körperliche Summenskala	Psychische Summenskala
*DASS*		
D: Depressivität (*n* = 10.614)	−0,150	−0,720
A: Angst (*n* = 10.676)	−0,105	−0,514
S: Stress (*n* = 10.446)	−0,098	−0,618
*MFHW*		
Wohlbefinden (*n* = 10.621)	0,342	0,485
*Schmerzbedingte Beeinträchtigungen bei*		
Alltag (*n* = 11.644)	−0,488	−0,271
Freizeit (*n* = 11.644)	−0,512	−0,306
Arbeitsfähigkeit (*n* = 11.644)	−0,477	−0,331

*DASS* Depression-Angst-Stress-Skalen, *MFHW* Marburger Fragebogen zum habituellen Wohlbefinden

Die psychische Summenskala wies hohe Beziehungen zu den Subtests der DASS aus. Höhere psychische Belastung war mit reduzierter psychischer Lebensqualität assoziiert (skalenabhängig zwischen 26 und 52 % gemeinsame Varianz). Die Beziehungen zur körperlichen Summenskala waren dagegen vernachlässigbar gering (1–2 % gemeinsame Varianz). Schmerzbedingte Beeinträchtigungen im Alltag, in der Arbeitsfähigkeit und in der Freizeit korrelierten höher mit der körperlichen als mit der psychischen Summenskala des VR-12. Habituelles Wohlbefinden war sowohl mit positiver psychischer als auch mit positiver körperlicher Lebensqualität mittelstark assoziiert.

### Beziehungen des VR-12 mit klassifizierten Schmerz- und Befindensparametern

Die Patienten mit Schmerzchronifizierungsstadium 1 hatten in beiden Summenskalen des VR-12 signifikant höhere Werte (bessere Lebensqualität) als die mit Stadium 2, und diese hatten signifikant höhere Werte als Patienten mit Chronifizierungsstadium 3. In Abhängigkeit von der Höhe des Schmerz-Schweregrads wurde eine reduzierte körperliche und psychische Lebensqualität beschrieben, die einzelnen Stufen unterschieden sich signifikant in den Werten.

Patienten mit Hinweis auf ausgeprägte Belastung durch klinisch relevante Depressivität, Angst oder Stress beschrieben insbesondere in der psychischen Summenskala des VR-12 eine deutlich geringere Lebensqualität. Tab. 4 zeigt die Werte der VR-12 Summenskalen für schmerzbezogene und psychische Merkmalsbereiche.

**Table 4:** 

	Körperliche Summenskala			Psychische Summenskala		
Merkmalsbereich	*M*	*SD*	*p*-Wert (Einzelvergleich)	*M*	*SD*	*p*-Wert (Einzelvergleich)
*Schmerzchronifizierung (MPSS)*	–	–	< 0,001	–	–	< 0,001
Stadium 1 (*n* = 835)	33,43	9,68	(1 > 2 > 3)	42,87	11,87	(1 > 2 > 3)
Stadium 2 (*n* = 4168)	29,65	8,91	1/2: *d* = 0,42	38,88	12,27	1/2: *d* = 0,33
Stadium 3 (*n* = 6641)	26,66	7,92	2/3: *d* = 0,36	36,05	12,58	2/3: *d* = 0,23
*Schmerzgrading (v. Korff)*	–	–	< 0,001	–	–	< 0,001
Grad 1 (*n* = 481)	38,65	8,81	(1 > 2 > 3 > 4)	46,36	10,66	(1 > 2 > 3 > 4)
Grad 2 (*n* = 1299)	35,18	8,37	1/2: *d* = 0,41	42,95	11,41	1/2: *d* = 0,30
Grad 3 (*n* = 2437)	30,43	7,96	2/3: *d* = 0,59	39,58	12,16	2/3: *d* = 0,28
Grad 4 (*n* = 7420)	25,58	7,43	3/4: *d* = 0,64	35,36	12,37	3/4: *d* = 0,34
*DASS‑D (Wert ≥* *10: Depressivität*)	–	–	< 0,001	–	–	< 0,001
Nein (*n* = 6729)	29,05	9,38		43,99	10,76	
Ja (*n* = 4794)	27,08	7,42	*d* = 0,23	28,70	8,99	*d* = 1,52
*DASS‑A (Wert ≥* *6: Angst)*	–	–	< 0,001	–	–	< 0,001
Nein (*n* = 6587)	28,84	9,33		42,44	11,92	
Ja (*n* = 5010)	27,40	7,63	*d* = 0,17	31,22	10,36	*d* = 1,00
*DASS‑S (Wert ≥* *10: Stress)*	–	–	< 0,001	–	–	< 0,001
Nein (*n* = 5537)	28,73	9,50		44,20	11,47	
Ja (*n* = 5828)	27,55	7,74	*d* = 0,14	31,12	10,08	*d* = 1,21

Die eingetragenen Einzelvergleiche in Klammern sind signifikant mit p < 0,05; paarweise Vergleiche darunter sind Effektstärken (d) zwischen Einzelgruppen

### Änderungssensitivität

Für 565 Patienten lagen Ergebnisse des DSF aus der Diagnostik vor Behandlungsbeginn und aus dem Verlaufsfragebogen des DSF zu einem Follow-up-Messzeitpunkt (Katamnese) vor. Dieser lag mindestens 13 Wochen nach der Diagnostik zu Behandlungsbeginn. Bei mehreren Follow-up-Erhebungen eines Patienten wurde der Messzeitpunkt gewählt, der zeitlich einer 6‑Monats-Nachmessung am nächsten lag.

Im Mittel lag die berücksichtigte Katamneseerhebung 20,8 Wochen nach der initialen Diagnostik (Median: 21,0 Wochen).

Bei Patienten, die ein stationäres Behandlungssetting erhalten hatten (*n* = 228), war das Zeitintervall mit 19,5 Wochen etwas kürzer als bei Patienten, die eine ambulante Behandlung erhalten hatten (*n* = 198, Zeitintervall: 20,8 Wochen). Am längsten war das Zeitintervall bei Patienten, die einer teilstationären Behandlung zugeführt worden waren (*n* = 139, Zeitintervall: 23,1 Wochen).

Tab. 5 fasst für die Gesamtgruppe die statistischen Kennwerte der Ausgangs- und Follow-up-Messung für die Schmerzangaben und die psychometrischen Verfahren zusammen und zeigt Angaben zur Änderungssensitivität der einzelnen Verfahren.

**Table 5:** 

	Ausgangsmessung		Follow-up Messung					
Verfahren	*M*	*SD*	*M*	*SD*	*t*-Wert	*p*-Wert	Korr	ES
*Schmerzintensität (0–10)*								
Momentane (*n* = 546)	5,66	2,08	4,95	2,37	7,07	< 0,001	0,45	0,33
Durchschnittliche (*n* = 546)	6,40	1,66	5,20	2,12	13,40	< 0,001	0,41	0,67
Größte (*n* = 546)	8,21	1,36	7,11	2,10	11,94	< 0,001	0,29	0,68
*Schmerzqualität*								
Affektive Dimension (*n* = 476)	5,41	3,69	3,76	3,61	9,31	< 0,001	0,44	0,42
*DASS*								
Depressivität (*n* = 563)	8,26	5,14	6,29	5,39	10,35	< 0,001	0,63	0,45
Angst (*n* = 563)	5,69	4,45	4,78	4,35	5,70	< 0,001	0,63	0,24
Stress (*n* = 497)	9,77	4,96	7,87	4,98	9,31	< 0,001	0,58	0,42
*MFHW*								
Wohlbefinden (*n* = 564)	11,20	8,00	16,80	9,08	13,21	< 0,001	0,31	0,60
*Beeinträchtigung (0–10) bei*								
Alltag (*n* = 546)	5,89	2,20	4,72	2,61	10,49	< 0,001	0,41	0,49
Arbeitsfähigkeit (*n* = 546)	7,12	2,22	5,52	2,95	12,77	< 0,001	0,39	0,61
Freizeit (*n* = 546)	6,74	2,22	5,12	2,80	13,49	< 0,001	0,40	0,67
*VR-12*								
Körperliche S.-Skala (*n* = 560)	29,09	7,76	32,99	10,33	10,07	< 0,001	0,52	0,51
Psychische S.-Skala (*n* = 560)	38,26	12,28	42,17	13,27	7,40	< 0,001	0,52	0,33

*DASS* Depression-Angst-Stress-Skalen, *MFHW* Fragebogen zum habituellen Wohlbefinden, *VR-12* Veterans Rand 12-item Health Survey, (*n*) Anzahl der Patienten, bei denen für das Verfahren beide Messungen vorlagen

*t*-Wert: Prüfgröße aus *t*-Test, *Korr* Korrelation zwischen den zwei Messungen, *ES* Effektstärke

Für alle Verfahren waren Unterschiede zwischen den zwei Erhebungen signifikant und in behandlungserwünschter Richtung (Abnahme der Schmerzstärke, Reduktion schmerzbedingter Beeinträchtigungen, Verbesserung des psychischen Befindens, Verbesserungen der gesundheitsbezogenen Lebensqualität).

Für die aktuelle Schmerzintensität, die affektive Dimension des Schmerzes, die schmerzbedingte Beeinträchtigung im Alltag, die negative Emotionalität (Depressivität, Angst, Stress) und die psychische Summenskala des VR-12 lagen die Größe der Veränderungen zwischen *d* = 0,20 und *d* = 0,49 und waren damit „gering“.

Für die Beurteilung der durchschnittlichen und größten Schmerzen, die Reduktion schmerzbedingter Beeinträchtigungen hinsichtlich Arbeitsfähigkeit und Freizeitaktivitäten, für habituelles Wohlbefinden und für die körperliche Summenskala des VR-12 waren Veränderungen „mittelstark“ (zwischen *d* = 0,50 und *d* = 0,80).

Die Berechnung der Änderungssensitivität als standardisierte Effektstärke nach Cohen (SES: Differenz der Mittelwerte der zwei Messungen geteilt durch die Standardabweichung der ersten Messung) ergab für die Summenskalen des VR-12 in der hier berichteten Analysestichprobe vergleichbare Werte wie die in Tab. 5 berichteten (körperliche Summenskala: *d* = 0,50; psychische Summenskala: *d* = 0,32).

In der körperlichen Summenskala des VR-12 waren die Veränderungen bei den teilstationären Patienten am größten (*d* = 0,74) und bei den stationären Patienten am geringsten (*d* = 0,42). Die Gruppe mit ambulantem Behandlungsanlass lag dazwischen (*d* = 0,51).

In der psychischen Summenskala des VR-12 waren Veränderungen bei Patienten mit ambulantem Behandlungsanlass sehr gering (*d* = 0,11) und für die stationären Patienten (*d* = 0,45) und teilstationären Patienten (*d* = 0,50) gering bis mittelstark.

**Figure 2:**
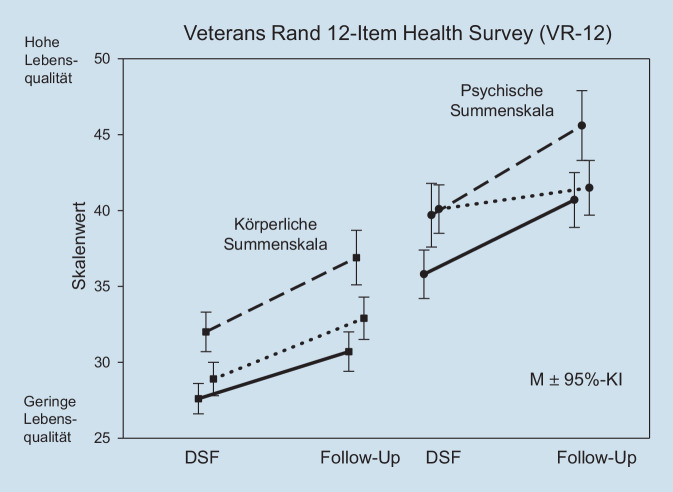

Abb. 2 zeigt die Summenskalenwerte des VR-12 bei Behandlungsbeginn und zur Follow-up-Messung. Dargestellt sind angepasste Mittelwerte mit Vertrauensintervall unter Bereinigung des Zeitintervalls (berücksichtigt als Kovariable) für die Patienten der drei Behandlungssettings.

Für die körperliche Summenskala lagen ein signifikanter Zeithaupteffekt (*p* = 0,002) und ein signifikanter Haupteffekt für „Behandlungssektor“ (*p* < 0,001) vor. Patienten mit stationärem Behandlungsanlass zeigten geringere (schlechtere) Werte als die mit ambulanter Behandlung (*p* = 0,02), und diese hatten geringere Werte als die teilstationären Patienten (*p* < 0,001). Die Interaktion Gruppe × Zeit war nicht signifikant (*p* = 0,20).

Für die psychische Summenskala des VR-12 ergab sich ebenfalls ein signifikanter Zeithaupteffekt (*p* < 0,001). Dieser ging aber ausschließlich auf Patienten mit stationärem und teilstationärem Behandlungsanlass zurück, wohingegen ambulant behandelte Patienten keine relevante Veränderung in der psychischen Summenskala beschrieben. Die Interaktion zwischen Zeit und Behandlungsanlass war entsprechend signifikant (*p* < 0,001).

## Diskussion

Unsere Analyse stellt den im DSF neu integrierten VR-12 in das Zentrum einer Auswertung zu Kennwerten und Güteeigenschaften des Verfahrens bei Patienten mit chronischem Schmerz, die einer interdisziplinären Schmerzbehandlung zugeführt wurden. Mit 11.644 Patienten konnte aus dem KEDOQ-Schmerz-Datensatz die größte bislang verfügbare Patientengruppe für eine solche Analyse berücksichtigt werden.

Die Verteilung der Summenwerte wich statistisch signifikant von einer Normalverteilung ab, dieser Effekt ist aber vor allem durch die sehr große Gruppe bedingt. Die Abbildung zur Verteilung der Werte veranschaulicht die hohe Annäherung an die Form einer Normalverteilung und zeigt, dass die Verteilung der beobachteten Werte in der körperlichen Summenskala für die ersten drei Standardabweichungen um den Mittelwert höchstens 1 % von einer idealen Normalverteilung abweicht. In der psychischen Summenskala ist dieser Wert mit maximal 3,5 % etwas höher. Die negative Kurtosis deutet für diese Summenskala auf etwas schwächer besetzte Randbereiche hin. Insgesamt erscheinen uns diese Abweichungen von einer Normalverteilung eher gering ausgeprägt und insgesamt unbedeutend.

Unsere Auswertung stellt erstmals statistische Kennwerte zu den Summenskalen des VR-12 vor, die nun zur Charakterisierung von Schmerzpatienten mit besonders geringer oder besonders hoher gesundheitsbezogener Lebensqualität im Vergleich zu anderen Patienten mit chronischem Schmerz verwendet werden können. Wird hierzu jeweils das untere und obere Quartil der Verteilung verwendet (vgl. [25]), so sind dies die Werte ≤ 22,1 oder ≥ 33,6 in der körperlichen Summenskala und die Werte ≤ 28,2 oder ≥ 46,7 in der psychischen Summenskala. Diese Werte sind (insbesondere für die körperliche Summenskala) gut vergleichbar mit den Werten zum SF-12, die für eine frühere Patientengruppe im KEDOQ-Schmerz-Datensatz 2017 berechnet wurden [13]. Tab. 6 zeigt den Vergleich.

**Table 6:** 

	VR-12 (vorliegende Analyse)		SF-12 (KEDOQ-Schmerz 2017)	
	Körperliche Summenskala	Psychische Summenskala	Körperliche Summenskala	Psychische Summenskala
*M (SD)*	28,2 (8,7)	37,6 (12,6)	29,4 (8,2)	40,6 (12,1)
*Korrelation der Skalen*	*r* = −0,024		*r* = 0,001	
*Hoher Wert für* ^a^ *Schmerzpatienten*	≥ 33,7	≥ 46,7	≥ 33,9	≥ 50,5
*Geringer Wert für* ^a^ *Schmerzpatienten*	≤ 22,1	≤ 28,2	≤ 23,4	≤ 30,7
*Korrelationen mit DASS*				
D (Depressivität)	−0,15	−0,72	−0,19	−0,70
A (Angst)	−0,11	−0,51	−0,15	−0,54
S (Stress)	−0,10	−0,62	−0,14	−0,64
*Korrelationen mit schmerzbedingten Beeinträchtigungen bei*				
Alltag	−0,49	−0,27	−0,47	−0,28
Freizeit	−0,51	−0,31	−0,47	−0,33
Arbeitsfähigkeit	−0,48	−0,33	−0,50	−0,31

^a^ Hoher Wert (hohe gesundheitsbezogene Lebensqualität)/geringer Wert (geringe gesundheitsbezogene Lebensqualität) entsprechend dem oberen/unteren Quartil der Werte aus der ausgewerteten Analysestichprobe von Patienten mit chronischem Schmerz; Daten aus KEDOQ-Schmerz (2017) [13] mit *N* = 7230

**Figure 3:**
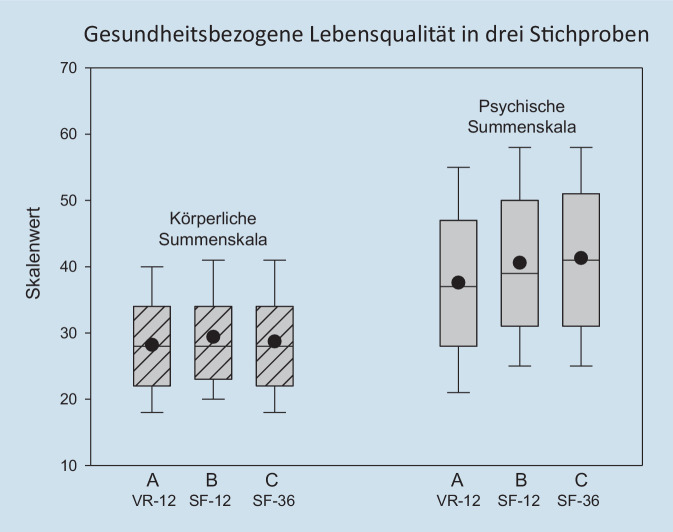

Abb. 3 veranschaulicht die Werteverteilungen aus beiden KEDOQ-Datenanalysen und ergänzt die Werte mit Daten einer Auswertung von Frettlöh et al. [7], die die Summenskalen des SF-36 bei Patienten mit chronischem Schmerz auswerteten. Die Verteilungseigenschaften der körperlichen Summenskala des VR-12 entsprechen danach gut denen der körperlichen Summenskala von SF-12 und SF-36. Die psychische Summenskala des VR-12 liegt bei bislang ausgewerteten Patienten des KEDOQ-Schmerz Kerndatensatzes dagegen etwa 3 Skalenpunkte unter denen des SF-12. Dies ist in Übereinstimmung mit den Befunden von Buchholz et al. [1] zum Vergleich des VR-12 mit denen des SF-12 bei orthopädischen und psychosomatischen Reha-Patienten. Für beide Gruppen war der Mittelwert der psychischen Summenskala des VR-12 geringer als der des SF-12 (Mittelwertsdifferenz bei orthopädischen Patienten 2,0 Punkte und bei psychosomatischen Patienten 4,0 Punkte). Demgegenüber erbrachte die körperliche Summenskala für beide Patientengruppen ähnliche Werte (Mittelwertsdifferenz bei orthopädischen Patienten 1,0 Punkte und bei psychosomatischen Patienten 0,8 Punkte).

Zur Praktikabilität des VR-12 (z. B. in Bezug auf den Anteil fehlender Werte) kann unsere Auswertung keine Aussage machen, da in unserem Auswertungskollektiv nur vollständige Datensätze berücksichtigt wurden. Buchholz et al. [1] berichten für den VR-12 bei Patienten einer psychosomatischen oder orthopädischen Rehabilitation itemabhängig zwischen 2,3 % und 5,2 % fehlende Angaben, für den SF-12 lag dieser Anteil zwischen 0,6 % und 7,2 %. Insgesamt ergaben ihre Auswertungen zwischen den zwei Verfahren keine deutlich erkennbaren Unterschiede bezüglich der Praktikabilität.

Die Zuverlässigkeit (Cronbachs Alpha) der Summenskalen des VR-12 ist mit *r*_*tt*_ = 0,782 (körperliche Summenskala) und *r*_*tt*_ = 0,842 (psychische Summenskala) akzeptabel bis gut [3]. Hierbei muss berücksichtigt werden, dass in die Berechnung der internen Konsistenz der Summenskalen ausschließlich die jeweiligen 6 Items eingingen, die entsprechend ihrer Zugehörigkeit zu den Skalen des VR-36 der körperlichen und psychischen Lebensqualität zuzuordnen sind. Da in die Berechnung der zwei Summenskalen im VR-12 jedoch jeweils alle 12 Items mit skalenabhängig unterschiedlichem Gewicht eingehen, müssen diese Angaben als Schätzung angesehen werden. Wegen dieser besonderen Skalenbildung wäre eine Retest-Reliabilitätsbestimmung (ohne zwischenzeitliche Intervention) angemessener. Solche Daten stehen jedoch nicht zur Verfügung. Die Auswertungen von Buchholz et al. [1] enthalten keine Reliabilitätsanalysen zum VR-12. Kazis et al. [19] berichten zum VR-12 zwar interne Konsistenzen (Cronbachs Alpha), sie berücksichtigen dabei aber ausschließlich die vier Skalen des VR-36, von denen jeweils zwei Items im VR-12 verwendet werden. Reliabilitätsberechnungen über Cronbachs Alpha liegen deshalb von Kazis et al. [19] für vier Subtests des VR-12 mit jeweils 2 Items vor. Ein Transfer auf unsere Ergebnisse ist daher nicht möglich.

Die zwei Summenskalen des VR-12 bilden unabhängige Aspekte der gesundheitsbezogenen Lebensqualität ab. Die Korrelation zwischen beiden Summenskalen beträgt *r* = −0,02. Sie ist damit ähnlich gering wie die aus dem KEDOQ-Schmerz-Datensatz 2017 für den SF-12. In dieser Analysestichprobe [13] korrelieren die Summenskalen des SF-12 mit *r* = 0,001. In der Analysestichprobe von Frettlöh et al. [7] korrelierten die Summenskalen des SF-36 etwas höher miteinander (*r* = −0,21, *N* = 8532), aber auch hier lag weniger als 5 % gemeinsame Varianz zwischen den Summenskalen vor.

Validitätshinweise zum VR-12 ergaben sich durch Beziehungen zu konstruktnahen psychometrischen Verfahren, aber auch durch Beziehungen zu schmerzbedingten Aktivitätseinschränkungen und durch Beziehungen zu Klassifikationen von Merkmalen.

Der VR-12 hat den Anspruch, gesundheitsbezogene körperliche und psychische Lebensqualität abzubilden. Entsprechend wären hohe Beziehungen zwischen der psychischen Summenskala und psychischen Belastungen zu erwarten. Dies wird in unseren Auswertungen in hohen Beziehungen zwischen der psychischen Summenskala und den Skalen der DASS deutlich, nicht aber für die körperliche Summenskala (siehe Tab. 6). Gerbershagen et al. [9] fanden in ihren Analysen zur gesundheitsbezogenen Lebensqualität bei Patienten mit chronischem Schmerz vergleichbare Beziehungen zwischen der psychischen Summenskala des SF-36 und Depressivität (Korrelation mit der Allgemeinen Depressionsskala ADS: *r* = −0,77) und Angst (Korrelation mit dem State-Trait-Angstinventar STAI: *r* = −0,67). Auch sie fanden keine deutlichen Beziehungen zwischen den psychischen Merkmalen und der körperlichen Summenskala des SF-36 (Depressivität: *r* = −0,14; Angst: *r* = −0,05).

Die körperliche Summenskala war deutlicher assoziiert mit schmerzbedingten Beeinträchtigungen. Dies ist in Übereinstimmung mit Auswertungen zu den Summenskalen des SF-12 für eine andere Patientengruppe im KEDOQ-Schmerz-Datensatz 2017 ([13]; siehe Tab. 6). Für alle drei Bereiche der schmerzbedingten Beeinträchtigungen sind im VR-12 und im SF-12 die negativen Beziehungen zur körperlichen Summenskala ausgeprägter als die zur psychischen Summenskala.

Sowohl die körperliche als auch die psychische Summenskala des VR-12 waren mit dem habituellen Wohlbefinden in plausibler Richtung assoziiert. Dies erklärt sich mit den Items des Marburger Fragebogens für Habituelles Wohlbefinden (MFHW), die inhaltlich sowohl körperliche Aspekte (z. B. Item 6: „Ich war mit meinem körperlichen Zustand einverstanden“) als auch psychische Aspekte beinhalten (z. B. Item 7: „Ich habe mich richtig freuen können“). Die Beziehung zur psychischen Lebensqualität war etwas ausgeprägter als die zur körperlichen Lebensqualität (24 % vs. 12 % gemeinsame Varianz). Auch im KEDOQ-Schmerz-Datensatz 2017 sind die Beziehungen der psychischen Summenskala des SF-12 zum habituellen Wohlbefinden ausgeprägter als die der körperlichen Summenskala. Der Anteil aufgeklärter Varianz ist dabei aber geringer als in der aktuellen Auswertung zum VR-12 (16 % vs. 4 % gemeinsame Varianz).

Die Summenskalen des VR-12 differenzieren Patientengruppen in Abhängigkeit vom Schweregrad der Schmerzen (Schmerzgrading) und dem Ausmaß der Schmerzchronifizierung (Schmerzstaging) sowie nach dem Ausmaß der psychischen Belastung.

Mit zunehmendem Schmerzchronifizierungsstadium (nach dem Mainzer Stadienmodell) zeigte sich in beiden VR-Summenskalen eine abnehmende gesundheitsbezogene Lebensqualität. Dies entspricht den Befunden von Frettlöh et al. [6] an einer Gruppe von 862 chronischen Schmerzpatienten aus der QUAST-Datenbank, die mit dem SF-36 untersucht waren. In Übereinstimmung sind die Befunde zudem mit der Ausgangslage von 1461 Patienten der QUAST-Datenbank, deren Behandlungserfolg in Abhängigkeit von der Schmerzchronifizierung untersucht wurde [11]. In Abhängigkeit vom steigenden Schmerzgrading nach von Korff zeigten sich im VR-12 abnehmende Werte in der körperlichen und psychischen Summenskala. Dies entspricht den Ergebnissen von Gerbersagen et al. [9], die die zwei Summenscores des SF-36 bei 1128 Patienten mit chronischem Schmerz mit den vier Graduierungsstufen nach v. Korff in Beziehung setzten. Zunehmendes Grading und Staging chronischer Schmerzen ist also mit abnehmender gesundheitsbezogener Lebensqualität verbunden, wobei die Beziehungen zur körperlichen Summenskala des VR-12 etwas stärker sind als die zur psychischen Summenskala.

Die Klassifikation der Patienten nach den Cut-off-Werten für Depressivität, Angst und Stress [23, 24] im DASS-Fragebogen ergab in der psychischen Summenskala des VR-12 plausible und sehr große Gruppenunterschiede. Hier zeigte sich eine geringere psychische Lebensqualität bei Patienten mit Hinweis auf ausgeprägte Belastungen in den erhobenen Bereichen. In der körperlichen Summenskala ergaben sich hier nur klinisch sehr geringe oder bedeutungslose Unterschiede. Dies kann als weiterer Hinweis auf die inhaltliche Validität des VR-12 bei Patienten mit chronischem Schmerz gewertet werden.

Die Auswertungen zur Änderungssensitivität zeigen, dass die Patienten über die Summenskalen des VR-12 mehrere Monate nach Behandlungsabschluss eine bessere Lebensqualität angeben als zu Behandlungsbeginn. Die Veränderungen waren mit einer Effektstärke von *d* = 0,33 in der psychischen Summenskala geringer als in der körperlichen Summenskala (*d* = 0,51). Das ist in Übereinstimmung zu Veränderungen, die in den Summenskalen des SF-36 bei 1229 Patienten mit chronischem Schmerz beobachtet wurde. Dort wurden aus dem QUAST-Schmerzregister Angaben zu Behandlungsbeginn mit denen des letzten verfügbaren Verlaufsfragebogens verglichen [11]. Zwischen beiden Messungen lagen etwa 14 Monate. Die Effektstärke der Verbesserung war für die körperliche Summenskala *d* = 0,41 und für die psychische Summenskala *d* = 0,23. Auch Buchholz et al. [1] zeigen in ihren Auswertungen zur Änderungssensitivität des VR-12 und SF-12 bei Patienten einer stationären orthopädischen oder psychosomatischen Rehabilitation, dass beide Messverfahren Veränderungen des Befindens zwischen Beginn und Ende der drei- (orthopädische Patienten) oder fünfwöchigen Behandlung (psychosomatische Patienten) abbilden. Das Ausmaß der Änderung ist in ihrer Patientengruppe allerdings in der psychischen Summenskala größer (VR-12: SES = 0,47; SF-12: SES = 0,54) als in der Körperlichen Summenskala (VR-12: SES = 0,22; SF-12: SES = 0,26), was insbesondere durch die deutliche Verbesserung der psychosomatischen Patienten bedingt ist. Die Patienten der hier berichteten KEDOQ-Analysegruppe beschreiben mehrere Monate nach Behandlungsabschluss in ihrer körperlichen gesundheitsbezogenen Lebensqualität die größere Besserung (SES = 0,50).

Zusammenfassend sprechen unsere Auswertungen dafür, dass mit dem VR-12 ein angemessener Ersatz zum lizenzpflichtigen SF-12 im Deutschen Schmerzfragebogen integriert wurde.

Dabei muss einschränkend angemerkt werden, dass unsere Auswertung ausschließlich auf Daten der Versorgungsqualitätssicherung (KEDOQ-Schmerz) beruht. Eine Berücksichtigung weiterer (psychometrischer) Verfahren zur theoriegeleiteten Analyse der konvergenten und diskriminanten Validität des VR-12 sowie ein direkter Vergleich mit dem SF-12 waren deshalb nicht möglich.

## Fazit für die Praxis

- Der VR-12 ist ein angemessener Ersatz zum SF-12 im Deutschen Schmerzfragebogen. Die Auswertung des Verfahrens ist allerdings nicht „von Hand“ möglich, sondern erfordert (wie beim SF-12) ein Auswertungsprogramm.
- Der VR-12 weist für Patienten mit chronischem Schmerz Kennwerte auf, die mit denen des SF-12 vergleichbar sind. Die Validität ist durch Beziehungen zu konstruktnahen psychometrischen Verfahren sowie zu schmerzbezogenen und psychischen Patientenmerkmalen gut belegt.
- Der VR-12 ist änderungssensitiv. Mit dem VR-12 kann der Erfolg einer Schmerzbehandlung im Hinblick auf die gesundheitsbezogene Lebensqualität aufgezeigt werden.
- Die große Stichprobe dieser Analyse erlaubt die Charakterisierung von Patienten mit chronischem Schmerz in solche, die im Vergleich zu anderen Schmerzpatienten eine besonders hohe, und solche, die eine besonders geringe Lebensqualität beschreiben.
- Die Verwendung des VR-12 im Deutschen Schmerzfragebogen und den Verlaufsfragebögen ist durch die Deutsche Schmerzgesellschaft bei den Autoren des Verfahrens gemeldet. Wer den VR-12 bei anderen Patientengruppen einsetzen möchte, kann die Nutzung registrieren lassen: https://www.bu.edu/sph/about/departments/health-law-policy-and-management/research/vr-36-vr-12-and-vr-6d/

## Supplementary Information






## Footnotes

1 Einige an KEDOQ-Schmerz beteiligten Zentren verwenden den VR-12 seit 2014. Ab 2016 wurde der VR-12 von allen Zentren verwendet. Patienten, die den VR-12 zwischen 2014 und 2016 ausgefüllt hatten (*N* = 185), wurden in die Analysegruppe aufgenommen.
