# Supplementary material for: Characteristic values and test statistical goodness of the Veterans RAND 12-Item Health Survey (VR-12) in patients with chronic pain: An evaluation based on the KEDOQ pain dataset
Source: Schmerz. 2021 Jul 19;36(2):109–20. [Article in German] doi: 10.1007/s00482-021-00570-5 (PMC8956556; doi:10.1007/s00482-021-00570-5)
Supplement: Supplementary file 1 [file 482_2021_570_MOESM1_ESM.pdf]

Elektronisches Zusatzmaterial: Gesundheitsbezogene Lebensqualität: VR-12

In diesem Fragebogen geht es um die Beurteilung Ihres Gesundheitszustandes. Ihre Angaben ermöglichen es nachzuvollziehen, wie Sie sich fühlen und wie gut Sie Ihre Alltagstätigkeiten ausüben können.

Bitte beantworten Sie jede Frage, indem Sie die Antwortmöglichkeit ankreuzen, die am besten auf Sie zutrifft. Wenn Sie sich bei der Antwort auf eine Frage unsicher sind, beantworten Sie diese bitte so gut wie möglich.

	aus-gezeichnet	sehr gut	gut	weniger gut	schlecht
**1.** Wie würden Sie Ihren Gesundheitszustand im Allgemeinen beschreiben?	☐	☐	☐	☐	☐

In den folgenden Fragen geht es um Tätigkeiten, die Sie vielleicht im Laufe eines normalen Tages ausüben. Sind Sie derzeit aufgrund Ihrer Gesundheit bei diesen Tätigkeiten eingeschränkt? Wenn ja, wie stark?

	ja, stark eingeschränkt	ja, etwas eingeschränkt	nein, überhaupt nicht eingeschränkt
**2. mittelschwere Tätigkeiten**, z.B. einen Tisch verschieben, staubsaugen, kegeln	☐	☐	☐
**3. mehrere** Treppenabsätze steigen....................................	☐	☐	☐

Hatten Sie in den vergangenen 4 Wochen aufgrund Ihrer **körperlichen** Gesundheit die folgenden Probleme bei der Arbeit oder bei anderen Alltagstätigkeiten?

	nie	selten	manchmal	meistens	immer
**4.** Ich habe weniger geschafft als ich wollte	☐	☐	☐	☐	☐
**5.** Ich konnte nur bestimmte Arbeiten oder andere Tätigkeiten ausführen	☐	☐	☐	☐	☐

Hatten Sie in den vergangenen 4 Wochen aufgrund **seelischer** Probleme die folgenden Schwierigkeiten bei der Arbeit oder bei anderen Alltagstätigkeiten (z.B. weil Sie sich niedergeschlagen oder ängstlich fühlten)?

	nie	selten	manchmal	meistens	immer
**6.** Ich habe weniger geschafft als ich wollte	☐	☐	☐	☐	☐
**7.** Ich konnte meine Arbeit oder andere Tätigkeiten nicht so sorgfältig wie sonst erledigen	☐	☐	☐	☐	☐

	überhaupt nicht	ein wenig	mäßig	ziemlich	sehr
**8.** Inwieweit haben Schmerzen in den vergangenen 4 Wochen Ihre Alltagstätigkeiten (im Beruf und zu Hause) beeinträchtigt?	☐	☐	☐	☐	☐

In diesen Fragen geht es darum, wie Sie sich in den vergangenen 4 Wochen gefühlt haben und wie es Ihnen ergangen ist. Bitte kreuzen Sie bei jeder Frage die Antwort an, die am besten beschreibt, wie Sie sich gefühlt haben.

Wie oft fühlten Sie sich in den vergangenen 4 Wochen…	immer	meistens	ziemlich oft	manchmal	selten	nie
**9.** ruhig und gelassen?	☐	☐	☐	☐	☐	☐
**10.** voller Energie?..	☐	☐	☐	☐	☐	☐
**11.** entmutigt und traurig?..	☐	☐	☐	☐	☐	☐

	immer	meistens	manchmal	selten	nie
**12.** Wie häufig haben Ihre körperliche Gesundheit oder seelischen Probleme in den vergangenen 4 Wochen Ihre Kontakte zu anderen Menschen (Besuche bei Freunden, Verwandten usw.) beeinträchtigt?	☐	☐	☐	☐	☐

Veterans RAND 12 Gesundheitsfragebogen ("VR-12: How to create VR-12 scales and PCS/MCS summaries" © 2014 by Trustees of Boston University, Dt. Version: Buchholz & Kohlmann 2015)
Hinweis: Der VR-12 ist Public Domain und kann nach Antrag bei L.E. Kazis kostenfrei verwendet werden. Nutzungsregistrierung: https://www.bu.edu/sph/about/departments/health-law-policy-and-management/research/vr-36-vr-12-and-vr-6d/request-access/

MUSTER
