# Supplementary material for: Characteristic values and test statistical goodness of the Veterans RAND 12-Item Health Survey (VR-12) in patients with chronic pain: An evaluation based on the KEDOQ pain dataset
Source: Schmerz. 2021 Jul 19;36(2):109–20. [Article in German] doi: 10.1007/s00482-021-00570-5 (PMC8956556; doi:10.1007/s00482-021-00570-5)
Supplement: Supplementary file 2 [file 482_2021_570_MOESM2_ESM.pdf]

Elektronisches Zusatzmaterial: Auswertung des VR-12 (nur möglich bei allen beantworteten Items)

1. den Antwortkategorien der 12 Items Zahlenwerte (1-3 oder 5 oder 6) zuordnen

Item l_01_gesund_allg (1=ausgezeichnet bis 5=schlecht)
Item I_02_mittelschwer (1=ja bis 3=nein)
Item I_03_treppen (1=stark eingeschränkt bis 3=nein)
Item I_04_koerperl (1=nein, nie bis 5=ja, immer)
Item I_05_dinge (1=nein, nie bis 5=ja, immer)
Item I_06_seelisch (1=nein, nie bis 5=ja, immer)
Item I_07_sorgfaltig (1=nein, nie bis 5=ja, immer)
Item I_08_behindert (1=überhaupt nicht bis 5=sehr)
Item I_09_ruhig (1=immer bis 6=nie)
Item I_10_energie (1=immer bis 6=nie)
Item I_11_traurig (1=immer bis 6=nie)
Item I_12_beeintrachtigt (1=immer bis 5=nie)

2. die Zahlenwerte umkodieren in Werte zwischen 0 und 100

l_01_gesund_allg (1=100) (2=85) (3=60) (4=35) (5=0)
l_02_mittelschwer (1=0) (2=50) (3=100)
l_03_treppen (1=0) (2=50) (3=100)
l_04_koerperl (1=100) (2=75) (3=50) (4=25) (5=0)
l_05_dinge (1= 100) (2=75) (3=50) (4=25) (5=0)
l_06_seelisch (1=100) (2=75) (3=50) (4=25) (5=0)
l_07_sorgfaltig_VR (1=100) (2=75) (3=50) (4=25) (5=0)
l_08_behindert (1=100) (2=75) (3=50) (4=25) (5=0)
l_09_ruhig (1=100) (2=80) (3=60) (4=40) (5=20) (6=0)
l_10_energie (1=100) (2=80) (3=60) (4=40) (5=20) (6=0)
l_11_traurig (1=0) (2=20) (3=40) (4=60) (5=80) (6=100)
l_12_beeintrachtigt (1=0) (2=25) (3=50) (4=75) (5=100)

3. Körperliche Summenskala (PCS) und Psychische Summenskala (MCS) durch Summation der gewichteten Einzelitems bilden und eine Konstante zur Summe addieren

Körperliche Summenskala (PCS) = Summe	Psychische Summenskala (MCS) = Summe
[(I_01_gesund_allg*0,07825238),<br>(l_02_mittelschwer*0,06506401),<br>(l_03_treppen*0,07483613),<br>(l_04_koerperl*0,07169783),<br>(l_05_dinge*0,07415414),<br>(l_06_seelisch*-0,05759826),<br>(l_07_sorgfaltig*-0,03226894),<br>(l_08_behindert*0,13397491),<br>(l_09_ruhig*-0,04241186),<br>(l_10_energie*0,02996896),<br>(l_11_traurig*-0,0533624),<br>(l_12_beeintrachtigt*0,00460968)]<br>plus die Konstante 21,0468597	[(l_01_gesund_allg*-0,00091593),<br>(l_02_mittelschwer*-0,03549865),<br>(l_03_treppen*-0,03157714),<br>(l_04_koerperl*-0,0251735),<br>(l_05_dinge*-0,02465223),<br>(l_06_seelisch*0,1266861),<br>(l_07_sorgfaltig*0,08087236),<br>(l_08_behindert*-0,02437137),<br>(l_09_ruhig*0,1094085),<br>(l_10_energie*0,06942713),<br>(l_11_traurig*0,1493789),<br>(l_12_beeintrachtigt*0,10857344)]<br>plus die Konstante 12,6620483
